# Influence of Type 2 Diabetes and Adipose Tissue Dysfunction on Breast Cancer and Potential Benefits from Nutraceuticals Inducible in Microalgae

**DOI:** 10.3390/nu16193243

**Published:** 2024-09-25

**Authors:** Domenico Sergi, Mattia Melloni, Angelina Passaro, Luca Maria Neri

**Affiliations:** 1 Department of Translational Medicine, University of Ferrara, Via Luigi Borsari 46, 44121 Ferrara, Italy; domenico.sergi@unife.it (D.S.); mattia.melloni@unife.it (M.M.); 2 Laboratory for Technologies of Advanced Therapies (LTTA)—Electron Microscopy Center, University of Ferrara, Via Luigi Borsari 46, 44121 Ferrara, Italy

**Keywords:** breast cancer, type 2 diabetes mellitus, insulin resistance, adipokines, microalgae, nutraceuticals

## Abstract

Breast cancer (BC) represents the most prevalent cancer in women at any age after puberty. From a pathogenetic prospective, despite a wide array of risk factors being identified thus far, poor metabolic health is emerging as a putative risk factor for BC. In particular, type 2 diabetes mellitus (T2DM) provides a perfect example bridging the gap between poor metabolic health and BC risk. Indeed, T2DM is preceded by a status of hyperinsulinemia and is characterised by hyperglycaemia, with both factors representing potential contributors to BC onset and progression. Additionally, the aberrant secretome of the dysfunctional, hypertrophic adipocytes, typical of obesity, characterised by pro-inflammatory mediators, is a shared pathogenetic factor between T2DM and BC. In this review, we provide an overview on the effects of hyperglycaemia and hyperinsulinemia, hallmarks of type 2 diabetes mellitus, on breast cancer risk, progression, treatment and prognosis. Furthermore, we dissect the role of the adipose-tissue-secreted adipokines as additional players in the pathogenesis of BC. Finally, we focus on microalgae as a novel superfood and a source of nutraceuticals able to mitigate BC risk by improving metabolic health and targeting cellular pathways, which are disrupted in the context of T2DM and obesity.

## 1. Introduction

Breast cancer (BC) represents a disease in which the cells of the mammary gland acquire the ability to escape the cell cycle control and go toward an uncontrolled proliferation, turning malignant [1]. This disease has become the most diagnosed malignancy in the world, overtaking lung cancer and representing the first cause of death from cancer in women [2]. In further support of its deleterious impact on health, BC in 2020 has caused 685 000 deaths globally despite achieving an annual mortality reduction of 2–4% per year due to early detection and novel therapies [3]. BC can be classified according to histological and molecular subtypes. The first subtypes identify the duct as the site of tumour origin or the lobes of mammary gland in case of BC [4]. In both types, the malignant cells can detach from the site of origin and invade surrounding organs and tissues generating metastases mainly in the liver, brain, lung and bone [5]. The molecular subtype classification divides BC based on the expression of hormone receptors such as oestrogen (ER) and progesterone (PR) receptors and human epidermal growth factor receptor 2 (HER2), which also affect the survival rates. In line with this, individuals diagnosed with a triple-negative BC present the lowest survival rate [4,5,6]. BC risk is shaped by a variety of factors including age, obesity, smoke, alcohol intake, radiation exposure, age of first menarche, pregnancy, breastfeeding, use of contraceptive, family history of BC and gene mutations with both high penetrance such as for BC Gene 1/2 (BRCA 1/2) and low penetrance, represented by single nucleotide polymorphisms [7]. Along with the aforementioned risk factors, Type 2 diabetes mellitus (T2DM) has also been proposed as a risk factor for BC [8]. T2DM is a non-communicable chronic metabolic disease representative of 95% of diabetes cases and with a worldwide increasing incidence, mostly in low- and middle-income countries [9]. According to the international diabetes federation data, the number of people with this global health emergency disease increased from 151 million in 2000 to 537 million in 2021, representative of 10.5% of adults aged 20–79 years, and this amount is predicted to further increase, reaching 643 million people in 2030 and 783 million in 2045 [10]. T2DM is a metabolic disorder characterised by chronic hyperglycaemia, which is a direct consequence of insulin resistance (IR), defined as a blunted response of tissues to the pancreatic hormone insulin and recognised as the hallmark of T2DM [11]. As a first physiological response to IR, pancreatic β-cells orchestrate a compensatory insulin hypersecretion, thereby leading to hyperinsulinemia. Hyperinsulinemia characterises prediabetes, a dysmetabolic state that precedes the onsets of T2DM and prevents the manifestation of hyperglycaemia. However, when the ability of the pancreatic β-cells to mount this compensatory response becomes defective, full-blown T2DM arises [12]. These hallmarks of T2DM, namely insulin resistance, hyperinsulinemia and hyperglycaemia, may represent the mechanistic link between T2DM and BC [13]. In addition, adipose tissue dysfunction, secondary to adipocyte hypertrophy typical of obesity, represents a shared pathogenetic mechanism between T2DM and BC. In this regard, the aberrant secretion of adipokines from the adipose tissue not only has been widely implicated in the pathogenesis of IR and T2DM [14] but is also involved in BC development and chemoresistance [15].

T2DM and obesity are modifiable risk factor for the onset of BC, and it may be controlled or delayed by adopting a healthy lifestyle that includes a balanced diet [16]. In this context, superfoods may represent a source of nutraceuticals, which, as part of a healthy dietary pattern, may contribute to improving metabolic health thereby lowering BC risk. In this regard, microalgae represent an innovative source of bioactive compounds, with potential benefits for human health, that can be implemented into the diet in the form of whole microalgae cell food, microalgae-based products or microalgae extracts [17]. Some of the active components whose synthesis can be induced in microalgae, in response to specific stimuli, are antioxidants (resveratrol and astaxanthin) and anti-inflammatory nutraceuticals (omega-3 polyunsaturated fatty acids) that may exert anti-diabetic effects [16,18]. Nowadays, different edible microalgae are approved for human consumption in Europe such as *Arthrospira platensis*, *Chlorella vulgaris*, *Chlorella pyrenoidosa* and *Chlorella luteoviridis*, but the list of the microalgae-based novel foods is continually being updated [19]. Recently, this list has been expanded, introducing as novel foods the microalgae *Odontella aurita*, *Ulkenia* sp., *Tetraselmis chui* and *Haematococcus pluvialis* [20,21]. Thus microalgae not only are a novel superfood with beneficial effects on metabolic health but also represent an eco-sustainable source of nutraceuticals, contributing to fighting climate change [22].

The aim of this review is to provide an up-to-date overview of the relationship between T2DM and adipose tissue disfunction on BC development also by dissecting the mechanisms by which hyperglycaemia, hyperinsulinemia and adipose tissue dysfunction contribute to BC pathogenesis. Furthermore, the review will provide insights into the role of microalgae-derived nutraceuticals, with a particular focus on polyunsaturated fatty acids and antioxidants other than vitamins, as tools to mitigate T2DM and thus BC development, also by improving metabolic health.

## 2. Epidemiology: Is There a Link between Type 2 Diabetes and Breast Cancer?

T2DM must not be considered as a mere metabolic disorder marked by an impairment in glucose homeostasis and hyperglycaemia. Indeed, T2DM is a multisystem disease and represents a risk factor for a plethora of diseases, including cardiovascular disease, neurodegenerative disorders [23] and several types of cancer [24,25,26], including BC [27,28]. In further support of the epidemiological link between T2DM and BC, women diagnosed with T2DM have an increased risk for BC, with this association being more marked when the data are adjusted for body mass index (BMI) and menopausal status [29]. According to this meta-analysis, which included Hispanic, Asian, American and European women, the relative risk (RR) increased from of 1.15 for women with T2DM to 1.22 after adjusting for BMI and menopausal status [29]. Additionally, women with pre-existing T2DM were reported to have an increased risk of being diagnosed with larger tumour size, late-stage tumours and invasive lymph nodes at the time of the diagnosis compared to women without T2DM, suggesting that T2DM have a prognostic value for BC aggressiveness [30]. However, despite the above evidence, the relationship between T2DM and BC remains controversial. For example, in the large-scale, prospective, UK Biobank cohort population-based study, 8182 cases of BC were identified with no overall association between T2DM and BC being detected (hazard ratio (HR) = 1.00, 95% confidence interval (CI) = 0.90–1.12) [28]. Nevertheless, an increased risk was observed in BC during the first five years following T2DM diagnosis (HR = 3.94, 95% CI = 3.23–4.80), but this association was shown to be reversed 10 years after T2DM diagnosis (HR = 0.66, 95% CI = 0.53–0.82), with a stronger inverse association 15 years after T2DM diagnosis (HR = 0.39, 95% CI = 0.30–0.50) [28]. However, although the reasons underpinning these discrepancies in the literature remain to be elucidated, it is undeniable that T2DM and the prediabetes provide a microenvironment that supports BC onset and progression.

## 3. Mediators Responsible for the Relationship between Type 2 Diabetes Mellitus and Breast Cancer and Their Molecular Mechanisms of Action

Hyperinsulinemia in the early stages of T2DM pathogenesis and hyperglycaemia, as a key metabolic manifestation of the disease, are plausible mediators of the link between T2DM and BC. Hyperglycaemia can support tumour progression via the increase in cancer cell proliferation, invasion, migration and inducing apoptotic resistance and chemoresistance [31]. With regard to the effects of hyperinsulinemia on BC pathogenesis, these are mediated by the powerful mitogen potential of insulin [32]. The effects of hyperglycaemia and hyperinsulinemia on BC pathogenesis are discussed below.

### 3.1. Hyperglycaemia

**Figure 1:**
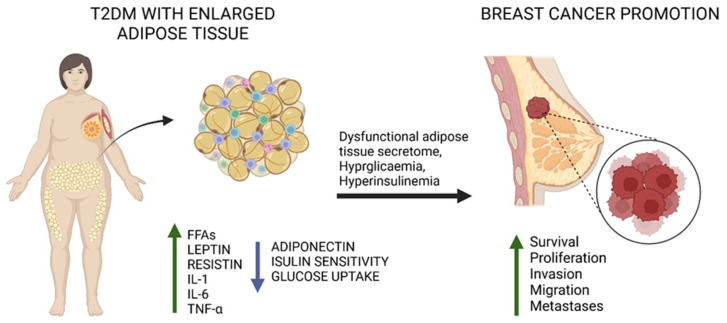
The impact of type 2 diabetes mellitus (T2DM) and hypertrophic, dysfunctional adipose tissue on breast cancer (BC) pathogenesis. The hallmarks of T2DM, namely hyperglycaemia and hyperinsulinemia, and the altered secretome of dysfunctional, hypertrophic adipocytes promote BC development and aggressiveness.

In a healthy organism and under physiological conditions, insulin promotes glucose clearance by inducing its uptake from metabolically active tissues, where it can be either catabolised or stored in the form of glycogen or fat. With regard to glucose catabolism and its use as an energy substrate, it can be metabolised via anaerobic glycolysis with the production of lactate or aerobic glycolysis [33]. Already in 1927, Warburg discovered that malignant cells, in comparison to normal cells, can increase their glucose uptake, producing at the same time more lactate via anaerobic glycolysis even in the presence of oxygen. The described phenomenon has been named after its discoverer and was termed the Warburg effect [34]. However, it was recently showed that malignant cells are able to call upon aerobic glycolysis and oxidative phosphorylation to sustain the fast rate of growth when in an aerobic microenvironment and to use anaerobic glycolysis in the core of the tumour due to its hypoxic microenvironment [35]. As described, a microenvironment enriched in glucose represents the perfect soil for the development of malignancies as in the case of BC (Figure 1). Indeed, numerous in vitro and in vivo studies investigating the effect of glucose on BC cells growth have revealed an increase proliferation rate also under hyperglycaemic condition [36,37,38,39,40]. The relationship between hyperglycaemia and BC encompasses several putative mechanisms. Hyperglycaemia can promote the epithelial-to-mesenchymal transition (EMT) [31], in which polarised epithelial cells undergo multiple biochemical changes that lead them to acquire a mesenchymal phenotype that leads to the degradation of the basement of the membrane [41] and enhances migratory and invasiveness capacity and resistance to apoptosis [42]. In this malignant process, hyperglycaemia can induce a matrix-specific EMT to promote the Warburg effect by increasing glucose uptake, hypoxia, lactate production and the expression of glycolytic enzymes and glucose transporters [43], which, in turn, results in tumour microenvironment acidification. In addition, the acidified tumour microenvironment has been shown to represent a condition related to increased oxidative stress, chronic inflammation and tumour metastasis [44]. The tumour microenvironment hypoxia, generated by hyperglycaemia, can lead to the upregulation of hypoxia-inducible factor 1 (HIF-1), increasing the synthesis and the stability of the α subunit of the homonym transcription factor, resulting in a driving force for tumour progression [45,46]. Indeed, HIF-1α has been shown to act as a vascularisation stimulator by inducing the expression of vascular endothelial growth factor (VEGF) [47,48]. However, other reports have suggested that the HIF activation can reduce tissue inflammation and improve glucose tolerance and insulin sensitivity [49], requiring further investigation to fully elucidate the hyperglycaemia-HIF axis. In an extensive in vitro study conducted on MCF-7 and MDA-MB-231 BC cells lines grown in hyperglycaemic conditions, it was shown that the latter cell line is more glucose-dependent than MCF-7 and that higher glucose concentration may upregulate the expression of glucose transporters (GLUTs) 1 and 3, which reflects the increased glucose uptake [50]. Furthermore, it has been shown that hyperglycaemia increases the migration speed of both BC cell lines via the reduction of cortical F-actin expression, chromatin condensation and the upregulation of both nuclear factor kappa-light-chain-enhancer of activated B cells (NF-kB) and mesenchymal markers such as vimentin and E-cadherin [50].

### 3.2. Hyperinsulinemia

After consuming a meal, the increase in glucose circulating levels is sensed by β-pancreatic cells. As a response, β-cells release insulin to stimulate its tissue target to uptake and metabolise glucose lowering its circulating levels and maintaining glycaemia in a tight range [51]. Insulin resistance dampens glucose uptake, leading to hyperglycaemia, which, as already described, is initially compensated by an insulin hypersecretory response by the pancreatic β-cells, leading to hyperinsulinemia, which precedes the onset of full-blown T2DM and characterises prediabetes [52]. Hyperinsulinemia has been associated with increased tumour risk and progression due to the mitogen effect of insulin (Figure 1). Indeed, it can promote cell proliferation by binding to its cognate receptor, leading to the activation of the downstream mitogen-activated protein kinase (MAPK) and phosphoinositide-3-kinase (PI3K)/protein kinase B (AKT) pathways [53]. When insulin binds to its cognate receptor, the cytosolic domain of the latter become phosphorylated, triggering the activation of the insulin pathway. The intracellular insulin signal transduction can, in turn, be divided into a metabolic and mitogenic branch [54,55]. In the metabolic one, the intracellular domain of the insulin receptor phosphorylates and activates the insulin receptor substrate 1 (IRS1), which leads to the activation of PI3K. Activated PI3K, as a consequence, catalyses the synthesis of phosphatidylinositol-3,4,5-triphosphate (PIP3) [56]. The increase in PIP3 amplifies the downstream signal through the activation of phosphoinositide-dependent kinase 1 (PDK1), which activates AKT [57]. AKT acts as a key node for the transduction of the insulin signal to different pathways via the activation of downstream targets involved in the modulation of glucose homeostasis (glycogen synthesis kinase 3, AKT substrate 160 kD), *de novo* lipogenesis and protein synthesis (mechanistic target of rapamycin complex) [58,59]. With regard to the mitogenic pathway, after insulin binding, the insulin receptor intracellular domain phosphorylates the adaptor proteins src homology and collagen protein, which, in turn, recruit the complex growth factor receptor-bound protein 2/son-of-sevenless [60]. This complex triggers the activation of the GTPase rat sarcoma (RAS), which, in turn, activates the rapidly accelerated fibrosarcoma (RAF) protein kinase. Finally, RAF activates its downstream effectors mitogen-activated protein kinase kinase 1/2 (MEK1/2) and extracellular signal-regulated kinase 1/2 (ERK1/2), known also as MAPKs, key enzymes in cell cycle regulation and cellular growth [61,62,63]. It has been shown that during IR, the metabolic pathway activated via PI3K/AKT/mTOR becomes impaired, but it is partially rescued by the compensatory hyperinsulinemia [32,64]. In contrast, there is evidence showing that during IR, the mitogenic pathway activated by insulin, responsible for cell proliferation stimulation and apoptosis inhibition, is not affected and possibly upregulated by hyperinsulinemia [65]. The ability of insulin to contribute to BC onset and progression is supported by the notion that BC tissue has a higher density of insulin receptor, up to six times, when compared with the mean receptor density in normal breast tissue [66]. Furthermore, it has been postulated that the overexpression of insulin receptors in BC cells may be a consequence of the downregulation and inactivation of the protein p53, which suppress the promoter activity of the insulin receptor gene, leading to its upregulation [62]. Furthermore, insulin stimulates the hepatic synthesis of the insulin-like growth factor 1 (IGF-1), a liver-derived growth factor that presents almost 50% amino acid sequence homology with insulin [67]. Unlike insulin, the circulating IGF-1 bioavailability is regulated by IGF-binding proteins (IGF-BPs) that hamper IGF-1 binding to its receptor, the IGF-1 receptor (IGF-1R). Insulin is able to enhance the circulating levels of free IGF-1 by downregulating the expression of IGF-BPs [68]. Thus, the increase in free IGF-1 circulating levels represents an additional mediator through which hyperinsulinemia promotes its mitogenic effects. In keeping with this, the activation of IGF-1R leads to an intracellular signal cascade that activates the MAPK pathway (responsible for increased cell proliferation), the PI3K pathway (responsible for apoptosis inhibition and the stimulation of protein synthesis), and the Janus kinase (JAK)/signal transducer and activator of transcription (STAT) pathway, mainly STAT3 (responsible for the transforming activity of IGF-1) [69]. Both IGF-1R and insulin receptor are composed of two α and two β subunits, but IGF-1R can be built in a hybrid form made up of one α- and β-subunit from the insulin receptor [70]. The hybrid form of IGF-1R and the high homology between IGF-1 and insulin allow, with a lower affinity, the binding of insulin to IGF-1R but not to the IGF-1R hybrid form; IGF-1 can bind to both IGF-1R and hybrid IGF-1R but, with a lower affinity, also to the insulin receptor [71]. Indeed, IGF-1 upregulation, stimulated by hyperinsulinemia, has been discovered to be associated with a greater risk of BC in a woman cohort of the UK Biobank, suggesting IGF-1 as a target for BC prevention [72]. Experiments in vitro have provided support for the relationship between hyperinsulinemia and BC, albeit with some discrepancies. In MCF-10A, a 24h insulin challenge (100 nM) increased cell proliferation by 184% [73]. On the contrary, insulin did not affect the proliferation rate of MDA-MB-231 and SK-BR-3 cell lines despite being able to induce the phosphorylation of its receptor by 380% in the MDA-MB-231 and by 35% for SK-BR-3 cells [73]. In another study conducted on the MDA-MB-231 BC cell line, it has been discovered that hyperinsulinemia (40 nmol/L for 48 h) increased the cellular proliferation as assessed by 3-[4,5-dimethylthiazol-2-yl]-2,5 diphenyl tetrazolium bromide and trypan blue exclusion assays. Furthermore, insulin can increase reactive oxygen species (ROS) concentration, which has been related to MDA-MB-231 migration, assessed via scratch-wound migration assay, and invasion assessed via 8 µm pore transwells coated with Matrigel [74]. Another study has investigated the proliferative effect of 25 nM insulin after 24h administration in MDA-MB-231 BC cell lines, concluding that the reported insulin concentration does not affect cell viability nor provide a proliferation stimulus when compared to the untreated control [75]. Furthermore, a study conducted in MCF-7 and MDA-MB-231 BC cell lines and in MCF-12A, a non-tumorigenic human breast epithelial cell line, concluded that 24 h treatment with insulin 50 nM promotes proliferation and migration on both the BC cell lines but not in MCF-12A [76]. These contrasting results underline the importance of further studies on the influence of a microenvironment characterised by hyperinsulinemia, also in combination with IGF-1 and hyperglycaemia, on BC cells proliferation and metastatic potentials.

## 4. Adipose-Tissue-Derived Adipokines as Drivers of Both Type 2 Diabetes and Breast Cancer

Adipose tissue is an intrinsic component of the mammary gland that has an important role in the communication of all components of the breast microenvironment apart from representing a site for energy storage and an endocrine organ. Adipose tissue overexpansion and dysfunction, as they occur in obesity, represent a further link within T2DM and BC. Indeed, adipocyte hypertrophy and dysfunction promote the dysregulation of adipose tissue secretome, which releases a wide array of mediators, termed adipokines, which contribute to both the pathogenesis of T2DM and BC [14,77]. These mediators include exosomes, cytokines, growth factors, miRNAs, and peptide hormones acting in both paracrine and endocrine fashion [78,79]. These adipokines are pivotal in the regulation of the crosstalk between the adipose tissue and other tissues, including the hypothalamus and the skeletal muscle [80,81]. Thus, adipose tissue secretome is implicated in modulating a variety of physiological processes, including energy metabolism, energy balance, immune response, cardiovascular function, reproduction and insulin sensitivity [82,83]. In light of this, it is not surprising that a disruption of the adipose tissue secretome contributes to insulin resistance, paving the way for the development of T2DM [84,85]. Beside their role in regulating energy and metabolic substrates homeostasis, fluctuations for example in the levels of specific adipokines, namely an increase in leptin and resistin and a decrease in adiponectin, have also been implicated in the pathogenesis of BC [86] (Figure 1).

### 4.1. Adiponectin

Adiponectin is an adipocyte derived protein hormone that exerts positive effects against inflammation, enhances fatty acids catabolism and improves insulin sensitivity [87,88]. The circulating levels of adiponectin in healthy subjects with a BMI lower than 25 has been shown to range from 5 to 30 µg/mL [89,90] with its levels dropping in obese individuals [91]. There are two types of adiponectin receptor known as adipoR1 and adipoR2, mainly expressed in skeletal muscle cells and hepatocytes, respectively [92]. Both receptors are able to activate the adenosine monophosphate-activated protein kinase (AMPK), p38 MAPK and peroxisome proliferation-activated receptor alpha (PPARα), thereby increasing cellular glucose uptake and fatty acid oxidation while decreasing anabolic pathways [93,94,95]. As anticipated, adipose tissue dysfunction leads to a decrease in leptin expression and circulating levels [96], an effect that lowers fatty acid catabolism, favouring the accumulation of intracellular fatty acid metabolites, such as diacylglycerol, fatty acyl CoA, and ceramides in tissues not suited for lipid storage [97,98], referred to as lipotoxicity. The intracellular accumulation of these lipotoxic metabolites promotes insulin resistance by activating serine/threonine kinases that impair the insulin pathway cascade [99]. Despite its metabolic role being established, a decrease in adiponectin has also been demonstrated in BC patients, suggesting that the effects of this hormone span beyond metabolic regulation [92,100,101]. Furthermore, adiponectin can act as a BC progression inhibitor via different mechanisms, including cellular proliferation inhibition, apoptosis and cytotoxic autophagy promotion [92,102]. Pham, DV et al., through in vitro assays conducted on the BC cell lines MDA-MB-231 and MCF-7, explained the mechanisms through which adiponectin can act as a breast tumour suppressor. They discovered that adiponectin can affect fatty acid synthesis and lipogenesis via sterol regulatory element-binding protein 1 (SREBP-1) downregulation. Furthermore, in the same BC cell lines, the authors observed that lipid raft disruption, secondary to lipid depletion, induced apoptosis in MCF-7 and MDA-MB-231 [103]. To further underline the potential involvement of adiponectin in the pathogenesis of BC, a meta-analysis has concluded that postmenopausal BC patients had significantly lower serum adiponectin than healthy subjects and that the levels of this adipokine may be used as a predictor to determine the patients that require more aggressive treatments and for survival rates [92].

### 4.2. Leptin

Another potential mediator linking adipose tissue dysfunction and BC is represented by leptin. Leptin is a 16 kDa globular peptide hormone, encoded by obese genes expressed in adipocytes, able to regulate appetite and metabolism by directly targeting the hypothalamus [104]. Adding to its main role in the regulation of energy homeostasis, leptin can also act on foetal and bone development [105,106], immune response [107] and the proliferation of many cell types, including breast cells [108,109]. Furthermore, it is well established that leptin levels directly correlate with adipocyte size in both women and men, with hypertrophic adipocytes leading, therefore, to an increase in plasma levels of leptin [110]. In keeping with this, leptin levels positively correlated with the BMI and HOMA-IR in all genders (T2DM males: 8.1 ng/mL of leptin, non-diabetic males: 3.8 ng/mL; T2DM females: 38.5 ng/mL, non-diabetic females 7.4 ng/mL) and to plasma insulin levels (r = 0.35, *p* = 0.007), suggesting the coexistence of IR and hyperleptinemia in subjects with T2DM [111]. High plasma levels of leptin can act as a pro-inflammatory stimulus, increasing the secretion of inflammatory molecules such as interleukin (IL)-6 and tumour necrosis factor (TNF)-α [112,113]. Furthermore, chronically elevated leptin in obese subjects may decrease the response of leptin receptors on pancreatic β–cells, generating leptin resistance and leading to an increase in insulin secretion [114]. Hyperinsulinemia, in turn, can further increase leptin levels, leading to a diabetogenic positive feedback loop [115]. Indeed, leptin has been proposed as an integrated biomarker for adiposity and IR in patients with T2DM [111,116]. Low levels of leptin and its receptor are expressed in healthy human mammary tissue, with their levels increasing in BC. In line with this, the upregulation of leptin and its receptor have been associated with early stages of carcinogenesis, particularly in patients with in situ ductal carcinoma compared with invasive breast carcinoma, suggesting that leptin and its receptor can promote the breast tumour progression [117]. Furthermore, leptin overexpression as previously reported in obese and T2DM subjects, has been correlated with increased risk of BC and larger and more advanced BC tumour [118,119]. In agreement with this, it has been reported that leptin in BC cells can induce the activation of multiple oncogenic pathways, including JAK/STAT3, ERK1/2 and PI3K, whose cascade culminates with the induction of genes involved in cell proliferation like cyclin D1 and c-myc or the activation of AKT/SREBP-2 pathway, respectively [109]. Furthermore, several in vitro studies have shown that leptin can induce EMT, metastases, and cell proliferation, as well as migration and invasion, in different BC cell lines such as MCF-7, MDA-MB-231 and SK-BR-3 [109,119]. Furthermore, different doses of leptin (10 ng/mL, 100 ng/mL and 1000 ng/mL) have been administrated to the 184B5 normal breast cell line and to the MCF-7, MDA-MB-231, and T47D BC cell lines, showing that leptin stimulates the proliferation of cancer cells but not of normal breast cells, further supporting the possibility that hyperleptinemia represents an effector through which obesity contributes to BC development [120].

### 4.3. Resistin

Human resistin is a 108-amino-acid adipokine involved in the pathogenesis of both T2DM and BC [121]. The term resistin has been related to the ability of this adipokine to promote IR by interfering with insulin signalling [122]. It has been reported that resistin concentration in human healthy subjects’ saliva ranges between 4.4 ng/mL and 7 ng/mL, with a significantly increased concentration in T2DM subjects, in whom it ranges from 6.9 ng/mL to 11.5 ng/mL [123] and positively correlates with IR [124,125]. Despite resistin being mainly produced by adipose tissue in rodents, in humans, the major production site is represented by monocytes, which, once differentiated into macrophages, the leading drivers of adipose tissue inflammation, represent the most abundant immune cells infiltrating this tissue in obese individuals [126]. Resistin has been reported to exert its effects by binding to the TLR4, which has been recognised as one of the four identified resistin receptors [127,128]. The binding of resistin to TLR4 triggers a signalling cascade, which, via the myeloid differentiation primary response adaptor protein and the interleukin 1 receptor-associated kinase (IRAK), culminates with the activation of NF-kB and MAPKs [129,130]. NF-kB, once activated, can translocate to the nucleus and induce the expression of pro-inflammatory cytokines, which are known to impair the insulin signalling transduction pathway and whose effects on BC will be discussed below [127,131]. In addition, resistin can upregulate the serine phosphorylation of IRS proteins through the activation of the serine kinases JNK and MAPK, which promotes IR by insulin signalling desensitisation [132]. Cancers, including BC, are not exempt from the deleterious effects of resistin which are mediated via the proinflammatory effects elicited by this adipokine. The relationship between resistin and BC is also supported by an increase in the circulating levels of this adipokine in individuals affected by BC [133,134]. Indeed, despite some discrepancies, clinical findings have reported higher resistin levels in BC patients with BMI > 25 compared with the health control group with BMI < 25, proposing this adipokine as a future potential therapeutic target [135,136]. The association between inflammation and tumorigenesis, particularly by promoting EMT, can represent the link between these two processes. Resistin can upregulate the expression of mesenchymal marker such as zinc finger E-box binding homeobox 1 (ZEB1), Zinc finger protein SNAI1 and 2 (SNAIL/SLUG), and Twist-related protein 1 (TWIST1) and downregulate the expression of epithelial markers (E-cadherine and claudin-1), thereby promoting the EMT transition responsible for the migratory and invasive potential of BC cells [137,138,139]. The mechanisms through which resistin can promote the EMT are not fully elucidated, but different studies have suggested that resistin, via its interaction with TLR4, can trigger NF-kB/STAT3 signalling, which results in the upregulation of EMT-related transcription factors and consequently BC promotion [140]. To further support the role of resistin in BC onset and progression, different studies have been conducted on the BC cells lines MCF-7 and MDA-MB-231, showing that in both of them, resistin increased the migration and invasion potential [138,141]. Thus, the epidemiological data supporting a positive relationship between resistin circulating levels and BC are also supported by mechanistic data. 

### 4.4. Pro-Inflammatory Mediators

Cytokines represent the major pro-inflammatory mediators released from the hypertrophic and dysfunctional adipose tissue (Figure 1). They can originate from adipocyte, preadipocyte and immune cells infiltrating the adipose tissue during obesity, particularly macrophages. Interleukins represent an integrant part of the immune response in which they function as an important communication mechanism between immune system cells, promoting their activation and differentiation, as well as proliferation, maturation, migration and adhesion [142,143,144]. The role of the immune system is not only to defend the host from infections or injuries but also to preserve the organism functionality in response to stress. As seen in the previous sections, over-nutrition can lead to adipose tissue expansion with adipocyte enlargement and the modulation of the adipokine secretory profile. In particular, pro-inflammatory mediators secreted from adipocytes, apart from contributing to local and systemic inflammation. promote the chemotactic monocyte extravasation from blood to adipose tissue. A key example of a mediator secreted by the adipose tissue and involved in promoting the chemotactic migration of monocytes is represented by Monocyte Chemoattractant Protein-1 [145]. Once monocytes have reached the adipose tissue, they can differentiate and contribute to the low-grade chronic inflammation typical of obesity by the release of cytokines which includes interleukins [146]. This low-grade chronic inflammation represents a key mediator of the metabolic aberrations triggered by obesity, including IR and therefore T2DM. Indeed, pro-inflammatory cytokines such as TNF-α and interleukins 1 and 6 have been widely implicated in the pathogenesis of T2DM by disrupting insulin signal transduction pathway [147]. Remarkably, besides contributing to T2DM development, the pro-inflammatory mediators secreted from the hypertrophic and dysfunctional adipose tissue have also been implicated in the pathogenesis of BC [148,149].

In this regard, IL-1β, apart from being upregulated in the plasma of individuals affected by T2DM [150] and being able to induce insulin resistance [151], it has been implicated in the pathogenesis of BC, as IL-1β circulating levels are higher in the serum of BC patients when compared to healthy subject’s serum [152,153,154]. Additionally, this cytokines contributes to BC initiation and progression, supporting proliferation, angiogenesis, EMT, invasion and metastasis [155,156]. Furthermore, in vitro studies have revealed that after IL-1β administration to the MDA-MB-231 and MCF-7 BC cell lines, their proliferation ratio increased, confirming the role of this cytokine in BC progression [156,157].

IL-6 represents a pleotropic cytokine whose synthesis take place in almost all cell types, including the cells of immune system [158]. IL-6 is able to exert physiological and pathophysiological functions, having a role in the regulation of metabolism, including insulin sensitivity, energy expenditure and lipid homeostasis, as well as endothelial function [159]. As described for IL-1β, IL-6 plasma levels are higher in individuals affected by IR [160] and T2DM [160,161,162,163]. IL-6 represents an additional shared pathogenetic mediator between T2DM and BC. In line with this, IL-6, upon binding to its receptor, activates the JAK/STAT3, RAS/MAPK and PI3K pathways, which play dominant roles in various types of cancer, including BC [164]. Indeed, the IL-6/JAK/STAT3 signalling pathway drives BC cell proliferation and invasiveness, promoting EMT, migration and metastasis while suppressing apoptosis [165]. Furthermore, STAT3 has been reported to enhance IL-6 signalling, thereby promoting a vicious inflammatory loop [165]. In further support of the involvement of IL-6 on BC development, in vitro studies have been conducted in MCF-7 and MDA-MB-231 BC cells. These studies have demonstrated that IL-6 induces growth and migration in both cell lines and EMT in MCF-7 but not in MDA-MD-231 due to the expression of high levels of vimentin, ZEB1 and SNAIL and low levels of E-cadherin [166,167,168,169]. Furthermore, clinical evidence suggests that IL-6 represents a prognostic factor for overall survival and that its plasmatic level upregulation is associated with poor prognosis in BC, mainly in triple-negative BC, for which therapeutic approaches are scarce compared with the other subtypes [170,171,172,173].

Not only interleukins but also pro-inflammatory cytokines such as TNF-α have been related to the induction of IR and T2DM [174], while also being implicated in the pathogenesis of BC [175]. From a metabolic point of view, TNF-α disrupts mitochondrial oxidative phosphorylation and impairs insulin-mediated glucose uptake by promoting IR [176]. TNF-α also derails adipocyte physiology. Indeed, once released, it is able to downregulate PPAR-γ, an essential transcriptional regulator of adipogenesis required also for mature adipocyte function, via different mechanisms. TNF-α, through the activation of NF-kB and activating protein 1, is able to impair the transcription of PPAR-γ mRNA and to impair the mRNA translation via the activation of MAPK cascade [177]. In addition, TNF-α has been recognised to reduce the expression of GLUT4, mainly expressed in skeletal muscles and adipocytes, and to promote the phosphorylation of IRS-1, mainly on the serine 307 residue, via the activation of the JNK pathway, playing a critical role on the onset of IR and T2DM [174]. In agreement with its metabolically deleterious effects, TNF-α circulating levels have been reported to be higher in obese individuals affected by T2DM compared to lean and metabolically healthy individuals [178,179]. Higher levels of TNF-α have been reported not only in subjects suffering from T2DM but also in the tumour microenvironment of BC patients, in which it covers an essential role in malignancy progression and metastasis [180,181]. Indeed, TNF-α is considered to be a pro-tumorigenic cytokine affecting BC cell proliferation, survival and EMT [175]. TNF-α can positively impact the proliferation of MCF-7 and MDA-MB-231 BC cell lines via the differential expression of critical assembly factors such as succinate dehydrogenase complex assembly factor 1 and the subunits involved in mitochondrial respiratory chain super-complexes [182]. In agreement with this, TNF-α induced the expression of the assembly factor for complex III, known as LYRM7, in MCF-7 and MDA-MB-231 BC cells, with these effects being mediated via the activation of the NF-kB signalling pathway. LYRM7, in turn, is involved in the maintenance of mitochondrial function and the regulation of survival and migration potentials of BC under inflammatory conditions. Indeed, defects in mitochondrial complexes can affect the mitochondrial membrane potential, impairing the synthesis of ATP while increasing ROS production [183]. Furthermore, LYRM7 is downregulated in BC patients compared to normal subjects, with the major reduction in the triple-negative BC subtype, indicating its relationship with the metastatic potential as well as survival outcome. However, the upregulation of LYRM7 expression, TNF-α mediated, has been reported to rescue the mitochondrial functions responsible, among other functions, for cell survival [183]. To further confirm the relationship between TNF-α and BC, it has been shown that the serum levels of TNF-α were higher in BC patients compared to healthy individuals and that they may also correlate with tumour stage [175].

## 5. Impact of T2DM on Breast Cancer Prognosis and Resistance to Chemotherapy

Patients with BC and pre-existing T2DM, who represent the 15% of BC patients, are not only exposed to a higher risk of all-cause mortality but also present a higher risk for cancer mortality compared to those without T2DM [184,185,186]. Furthermore, different studies have reported that patients with both T2DM and BC have lower overall survival and disease-free survival compared to those without T2DM [187,188,189,190]. However, after adjustment for BMI, tumour-node metastasis stage and stratification of age, the overall survival and disease-free survival did not significantly differs between BC patients with and without T2DM, suggesting obesity, old age, diabetic treatment and concomitant diseases as confounding factors on the influence of T2DM on BC [191]. In addition to this controversial, complex and not fully understood nexus between T2DM and BC, another layer of complexity is given by antidiabetic treatments. The administration of molecules able to sensitise the cellular response to insulin, reducing IR, such as metformin, are able to impact BC prognosis [46]. Metformin, in fact, can exert an antitumor effect via cell cycle alteration consisting of the AMPK-mediated inhibition of mTOR and inhibiting the tyrosine kinase receptors that trigger PI3K/AKT/NF-kB and RAS/RAF/MEK/ERK signalling. Furthermore, metformin can inhibit cancer invasion and migration, suppressing the EMT process by the downregulation of the transcription factors SNAIL, ZEB and TWIST [192,193]. The deleterious effect of T2DM on BC also relies on its impact upon chemotherapy efficacy and chemoresistance [44,188,194]. For example, hyperglycaemia can trigger the degradation of the homeodomain-interaction protein kinase 2, which mediates the p53 dependent apoptotic pathway in cancer cells, upregulating the expression of the mutant p53. Indeed, mutant p53 has been positively correlated with the expression of the P-glycoprotein, known as a protein biomarker of chemoresistance due to its drug efflux pump action [44]. Furthermore, hyperglycaemia may also promote chemotherapy resistance. The chemoresistance triggered by hyperglycaemia may rely on the activation of fatty acid synthase (FAS) and ceramide synthesis [195]. In this regard, the rise in glycolytic fluxes increases the availability of malonyl CoA, which is used by FAS to synthetise fatty acids, of which palmitic acid is the most abundant. Palmitic acid, in turn, represents the precursor of ceramide synthesis [196]. In support of the role of FAS and ceramide synthesis in mediating chemoresistance, hyperglycaemia was able to decrease the sensitivity to the chemotherapeutic agents 5-fluorouracil, doxorubicin and paclitaxel [195] in cells overexpressing FAS, including the BC cell lines MCF-7 and T47D [195]. Additionally, it was sufficient to inhibit FAS or ceramide synthesis in order to abrogate the chemoresistance elicited by hyperglycaemia [195,197]. However, not only hyperglycaemia but also adipokines can provide chemoresistance in BC. In this regard, resistin can confer chemoresistance in MCF-7 and MDA-MB-231 BC cell lines via autophagy induction, and it is able to suppress apoptosis promoted by doxorubicin [198]. Not only resistin but also leptin can exert chemoresistance. Indeed, leptin can activate NF-kB signalling, increasing BC cell survival despite the co-administration of chemotherapeutic drugs [199]. Furthermore, leptin acts as a potent mitogen [200] and possesses antioxidant activity, being able to enhance silent information regulator sirtuin 1 (SIRT1) protein levels and prevent ROS generation [201], which can ensure cancer proliferation and survival also when cells are treated with pro-oxidant drugs such as cisplatin [202]. Indeed, MCF-7 cells chronically exposed to leptin and treated with cisplatin showed an increased growth rate compared to controls [202]. In the previous sections, we have also discussed the upregulation of interleukins in T2DM patients and specifically of IL-1β, which has been reported to increase the resistance of MCF-7 (ER+ cells) to tamoxifen [203], doxorubicin [204] and cisplatin [205]. Additionally, this cytokine can induce the expression of sequestosome 1 gene not only in hormone receptor negative BC, in which it is essential for survival, but also in hormone receptor positive BC, in which it has a role in HR-independent survival and chemotherapeutic resistance [206]. Furthermore, IL-1β can stimulate IL-6 production in tissue transglutaminase 2-overexpressing MCF-7 cells through an NF-kB-, PI3K- and JNK-dependent mechanism, which represents a critical survival signal for BC stem cells, leading to in vivo tumorigenesis and drug resistance [207,208]. In addition, IL-6, recognised to be involved in tumour growth, metastasis and angiogenesis, has also been discovered to induce chemoresistance in BC cells. Specifically, in a study conducted on MDA-MB-231 BC cells, IL-6 protected malignant cells from chemotherapy-induced cytotoxicity and apoptosis via the involvement of the HIF-1, which regulates vital biological processes of tumour survival and progression [209].

## 6. Nutraceuticals Inducible in the Superfood Microalgae as Tools to Counter the Pathophysiological Mechanisms Associated with Type 2 Diabetes Mellitus and Adipose Tissue Dysfunction: The Potential Influence on Breast Cancer

The impact of T2DM and adipose tissue dysfunction on BC onset and progression may be modulated by nutrients and nutraceuticals. 

Microalgae represents a natural factory that may be modulated under specific stimuli such as light, pH, osmolality and temperature to enlarge the production of nutrients naturally present in microalgae but in small quantities [210,211]. Indeed, in addition to their high protein content and well-balanced amino acid profiles, microalgae possess the capability to synthetise a wide array of nutraceuticals known for their positive effects on human metabolic health like bioactive molecules with anti-oxidant, immunomodulatory, anti-diabetic and anti-tumour properties [17,212].

The beneficial effects of microalgae on human metabolic health in humans have already been extensively reviewed elsewhere [16,18,213]. In support of the metabolically beneficial effects of microalgae, it has been reported that *Arthrospira platensis*, commonly known as spirulina, can reduce body weight and body mass index when supplemented for more than 12 weeks. This advocates its potential implementations as an adjuvant therapy for obesity. The effects of spirulina are not limited to favouring body weight loss but may also impact upon glycaemic control. In line with this, spirulina biomass or extracts have been demonstrated to be able to improve glucose homeostasis when ingested at doses ranging from 2 to 6 g per day [16]. Nevertheless, in a randomised controlled double-blind clinical trial involving both healthy individuals and patients with T2DM, the intake of 20 g per day of spirulina sauce (containing 2 g of spirulina) or a placebo over a two-month period did not result in a clinically meaningful reduction in glycated haemoglobin (HbA1c) levels or fasting blood glucose. However, the authors observed a significant reduction in insulin levels, HOMA-IR, triglycerides, total cholesterol, low-density lipoprotein, appetite and oxidative stress [214].

Not only spirulina but also other microalgae have been studied for their effects on metabolic health [215]. However, some of these studies did not demonstrate significant improvements in glycaemic control, lipid profiles, or anthropometric indices in patients with type 2 diabetes [216]. Nevertheless, clinical trials assessing the efficacy of microalgae extracts in improving metabolic health by ameliorating hyperglycaemia, hyperinsulinemia and adipose tissue-derived adipokines are still limited; therefore, further research is warranted in order to address these gaps in the literature.

**Figure 2:**
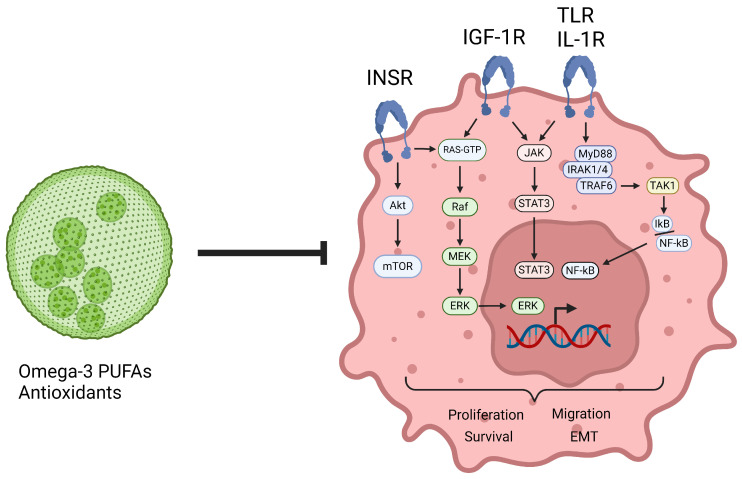
Microalgae-derived nutrients as tool to mitigate the impact of type 2 diabetes mellitus (T2DM) on breast cancer (BC). The microenvironment typical of T2DM and prediabetes, characterised by hyperglycaemia and hyperinsulinemia, promotes the activation of anabolic pathways. Insulin, upon binding to its receptor, triggers the activation of the AKT-mTOR axis, which enhances BC cell growth and proliferation. Furthermore, hyperinsulinemia triggers also the activation of the insulin mitogenic pathway, which leads to the nuclear translocation of ERK, leading to the upregulation of pro-survival and proliferation genes. Not only hyperinsulinemia but also IGF-1 activates the signalling cascade, which leads to the nuclear translocation of ERK. Furthermore, IGF-1 promotes the activation of the JAK/STAT3 pathway involved in epithelial to mesenchymal transition (EMT), BC growth, invasiveness, migration and metastasis promotion. The activation of TLR and interleukin receptors (reported in the figure only as IL-1R) culminates with the activation of the JAK/STAT3 pathway and the nuclear translocation of NF-kB via the MYD88/IRAK1-4/TRAF6/TAK1/IkB cascade, which promotes EMT and acts as a pro-survival stimulus in BC [137]. Nutrients, naturally present or inducible in response to environmental stressors in microalgae approved for human consumption, are able to counteract the aforementioned malignant pathways, promoted by T2DM and dysfunctional adipose tissue secretome, in BC cells. AKT, protein kinase B; ERK, extracellular signal-regulated-kinase; IGF-1, insulin-like growth factor-1; IGF-1R, insulin-like growth factor-1 receptor; IkB, NF-kappa-B inhibitor; INSR, insulin receptor; IRAK, interleukin 1 receptor-associated kinase 1-4; JAK, Janus kinase; MEK, mitogen-activated protein kinase kinase; mTOR, mechanistic target of rapamycin complex; MYD88, myeloid differentiation primary response protein 88; NF-kB, nuclear factor kappa-light-chain-enhancer of activated B cells; PPAR-α/γ, peroxisome proliferator-activated receptors α/γ; PUFAs, polyunsaturated fatty acids; RAF, rapidly accelerated fibrosarcoma; RAS, rat sarcoma; STAT3, signal transducer and activator of transcription 3; TAK, transforming growth factor-β-activated kinase; TLR, toll-like receptor; TRAF, TNF receptor-associated factor.

Examples of nutrients and nutraceuticals with a promising role in this context are represented by omega-3 polyunsaturated fatty acids (PUFAs) as well as antioxidants including polyphenols [18,217] (Figure 2).

### 6.1. Polyunsaturated Fatty Acids

Different studies have investigated the effect of PUFAs on metabolic health, specifically omega-3 fatty acids such as eicosapentaenoic acid (EPA) and docosahexaenoic acid (DHA), reporting their potential beneficial role in improving glycaemic control and insulin sensitivity in metabolically active tissues [218,219]. However, despite the beneficial effects of omega-3 PUFAs on insulin sensitivity remaining controversial [220], there are studies proposing their integration into the diet, specifically DHA, for their potential to inhibit many of the pro-tumorigenic pathways activated in individuals suffering from T2DM [218,221,222]. Indeed, omega-3 PUFAs can downregulate the expression of pro-inflammatory genes by directly interacting with nuclear receptors such as PPAR-α/γ and mitigating the activation of NF-kB and its translocation into the nucleus by dampening the phosphorylation and degradation of the NF-kB inhibitor [221,223]. The ability of omega-3 fatty acids to counter the activation of NF-kB signalling may also have important implications on BC, given the involvement of this pathway on BC pathogenesis [224,225]. Furthermore, it has been reported that omega-3 PUFAs can increase the circulating levels of adiponectin in patients with T2DM [226], which, as already described, is an adipokine inversely related to BC risk. In addition, EPA and DHA can compete with the omega-6 PUFA arachidonic acid (AA) for lipoxygenase and cyclooxygenase enzymes, promoting the synthesis of anti-inflammatory eicosanoids such as resolvins and protectins, which protects DNA and lipids from oxidative damage [227,228,229,230]. In overweight and obese subjects, the accumulation of AA in adipose tissue can contribute to the low-grade inflammation that can promote BC development and progression [231]. Omega-3 PUFAs have also been tested alone or in combination with conventional BC treatments, in order to assess their effects on BC prevention or therapeutic potential [232,233]. In this regard, a meta-analysis, conducted on 913 articles for a total of 130,365 Asian patients, has been performed to assess the protective effect of omega-3 PUFAs intake, from dietary fish consumption, against BC. Although the study presented some limitations, the authors concluded that the high consumption of fish, rich in omega-3 PUFAs, exerts a protective role against BC risk, with an odds ratio of 0.80 [234]. The protective role of omega-3 PUFAs has also been investigated in a systematic review based on studies conducted in murine models. In animal models, the omega-3 PUFA supplementation, in combination with chemotherapy or other BC traditional therapies, can act as an important coadjutant, reducing tumour growth and weight in the first fifteen days after tumour induction. Always in line with this, isolated omega-3 PUFA induced a tumour growth reduction in transgenic and chemically induced BC murine models after up to 300 days of supplementation despite the optimal dose remaining unclear [235].

### 6.2. Antioxidants Other than Vitamins

Antioxidants, known for their ROS scavenging activities, are representative of a plethora of molecules that comprise PUFAs and vitamins but also flavanols such as quercetin, phenols such as caffeic acid, carotenoids such as β-carotene, astaxanthin, fucoxanthin and many others, as we have previously reported [18]. Antioxidants such as β-Carotene [236], astaxanthin [237], lutein, quercetin, apigenin, caffeic acid and kaempferol [213] can counteract IR, preventing the onset of full-blown T2DM through different mechanisms. Indeed, the aforementioned antioxidants can promote the nuclear translocation of the nuclear factor erythroid 2-related factor 2 [238,239,240,241], which, in turn, mediates the expression of antioxidant genes [242]. Furthermore, these antioxidants can promote the inhibition of NF-kB signalling [236,237,241,243] and, as a consequence, the secretion of cytokine such as IL-1β. In addition, antioxidants can inhibit the phosphorylation of MAPKs, promoting at the same time anticancer effects [244]. In line with this, the carotenoid astaxanthin has been reported to inhibit the proliferation and migration of MCF-7 and MDA-MB-231 BC cells compared to the non-tumorigenic MCF-10A cell line [245]. Indeed, astaxanthin, only in BC cells, but not in normal epithelial breast MCF-10A cells, inhibits migration and reduces cell number, induces apoptosis and blocks the cellular proliferation [245]. In a recent systematic review and meta-analysis, based on 20 188 participants and 7608 cases, it was reported that each 10 μg/dL increase in circulating total carotenoids, α-carotene, β-carotene, and β-cryptoxanthin was respectively associated with 2%, 22%, 4% and 10% lower risk of BC [246]. Another study has investigated the relation between plasma carotenoids and risk of BC, concluding that higher concentrations of α-carotene, β-carotene, lycopene and total carotenoids were associated with lower risk of BC [247]. Polyphenols have also shown potential antitumor activity, also against BC, affecting different molecular targets such as kinases, pro-apoptotic proteins and enzymes that regulate energy metabolism, as well as signalling pathways linked to cell proliferation [248].

#### Polyphenols

Recent reviews have resumed the potential of polyphenols in IR and T2DM, their metabolic effect on BC cells and their role in modulating BC cells apoptosis and proliferation [249,250,251]. Indeed, it has been reported that the polyphenol resveratrol can increase IRS1 protein level and the GLUT4-mediated intracellular glucose uptake in insulin resistant mice by improving lipid metabolism and dampening lipotoxicity [249]. Furthermore, resveratrol is able to restore AMPK activity in IR rodents and trigger SIRT1 activity and expression. The effects of other polyphenols such as curcumin, quercetin, catechins, isoflavones, caffeic acid and others on IR and T2DM, are extensively reported elsewhere [249]. Regarding the effects of polyphenols on BC metabolism, it has been shown that the flavonoid genistein reduced glucose uptake in MCF-7 and MDA-MB-231 BC cells, while resveratrol suppressed glucose uptake and glycolysis in T47D BC cells [250]. This effect also held true for quercetin, which inhibited glucose uptake, in a dose-dependent manner, in MCF-7 and MDA-MB-231 BC cells and the consequent lactate production [250]. Cancer cells present a high consumption rate of glucose due to the overexpression of almost all glycolytic enzymes, which contributes to sustain proliferation, survival, invasion and metastasis [252]. However, polyphenols such as luteolin, kaempferol, rutin and gallic acid are able to inhibit the glycolytic pathway and may represent potential adjuvants in cancer therapy [250]. Taken all together, the above nutrients and nutraceuticals through their different mechanisms of action are able to improve metabolic health and glucose metabolism with consequent beneficial effects on the pathogenesis of T2DM. In light of this and considering the relationship between poor metabolic health and BC onset and progression, it is tempting to speculate that nutritionally targeting T2DM and its pathogenetic mechanisms may also positively impact BC tumorigenesis, progression and improve the prognosis in BC patients.

## 7. Conclusions

In conclusion, T2DM represents a risk factor for the onset and progression of BC. In particular, this relationship appears to be driven by hyperglycaemia and hyperinsulinemia, with the latter particularly characterising prediabetes. An additional player in this context is represented by adipose tissue dysfunction, which is a key driver of insulin resistance, the hallmark of T2DM. Remarkably, adipose tissue dysfunction and particularly the alteration of its secretome, has also been implicated in BC pathogenesis. Thus, the hypertrophy and dysfunction of adipocytes that lead to an aberrant secretion of adipokines and pro-inflammatory cytokines represent a shared pathogenetic mechanisms between T2DM and BC. Taken together, hyperinsulinemia, diabetic hyperglycaemia and the impaired release of adipokines and inflammatory cytokines from dysfunctional adipocytes all contribute to BC risk, development, invasiveness, metastatic potential, chemoresistance and poor BC prognosis. Nutraceuticals typically occurring or inducible in microalgae approved for human consumption may be a promising tool to lower BC risk and prognosis by improving the metabolic profile of type 2 diabetic individuals. Indeed, nutraceuticals such as omega-3 PUFAs, carotenoids and phenolic compounds have been shown to improve insulin sensitivity in T2DM subjects via direct ROS scavenging activity, the inhibition of inflammatory pathways, the improvement in lipid metabolism and the induction of the antioxidant defence system. In conclusion, microalgae-derived nutraceuticals may dampen BC development, retrieving in part the metabolic aberrations of type 2 diabetic individuals and improving adipocyte dysfunction. Furthermore, these nutraceuticals, due to their safety profile, may be proposed as coadjutants during the administration of common BC therapies. However, whether their ability to counter BC pathogenesis relays on their metabolically beneficial effects on T2DM warrants further investigations.
